# Development and psychometric evaluation of the DysPalKT for assessing nurses’ knowledge of dyspnoea care in patients with cancer receiving palliative care

**DOI:** 10.1186/s12912-026-04540-z

**Published:** 2026-03-14

**Authors:** Johanna Kero, Jaana-Maija Koivisto, Minna Stolt, Elina Haavisto

**Affiliations:** 1 Department of Health Sciences/Nursing, Faculty of Social Sciences, Tampere University, Arvo Ylpön katu 34, Tampere, 33520 Finland; 2 Department of Public Health, Faculty of Medicine, University of Helsinki, Helsinki, Finland; 3 Department of Nursing Science, University of Turku, Turku, Finland; 4 The Wellbeing Services County of Satakunta, Pori, Finland; 5 Tampere University Hospital, Tampere, Finland; 6 Turku University of Applied Sciences, Turku, Finland

**Keywords:** Palliative care, Dyspnoea, Knowledge test, Nurses’ knowledge, Psychometric properties

## Abstract

**Background:**

Dyspnoea is one of the most prevalent and distressing symptoms among patients with advanced cancer receiving palliative care. Nurses, particularly in non-specialist settings, often report insufficient preparedness in managing dyspnoea. Despite its clinical relevance, existing tests inadequately assess nurses’ knowledge of dyspnoea care. This study aimed to develop and evaluate the Knowledge of Dyspnoea Care in Patients with Cancer Receiving Palliative Care (DysPalKT) to address this gap.

**Methods:**

The DysPalKT was developed through a multi method approach including literature synthesis, expert panel validation, and pilot testing. A cross-sectional psychometric evaluation was conducted with 84 registered nurses working in general inpatient wards across seven district hospitals in Finland. Psychometric properties were analysed using the Kuder-Richardson Formula 20 (KR-20) and Rasch modelling.

**Results:**

The final test comprised 16 items across three domains. Content validity was supported by expert evaluations (I-CVI ≥ 0.83). However, KR-20 (0.306) indicated weak internal consistency, and Rasch analysis revealed low person separation and multidimensionality. Most items were too easy, limiting the test’s ability to differentiate between knowledge levels.

**Conclusions:**

The DysPalKT shows promise for assessing nurses’ knowledge of dyspnoea care in palliative care settings, but further refinement is needed. Increasing item difficulty and expanding the item pool may improve its reliability and discriminatory power. These improvements could enhance the knowledge test’s ability to identify learning needs, thereby supporting the design and targeting of future educational interventions in non-specialist palliative care.

**Supplementary Information:**

The online version contains supplementary material available at 10.1186/s12912-026-04540-z.

## Background

Palliative care (PC) plays a vital role in enhancing the quality of life for patients and their families facing life-threatening illnesses by alleviating suffering through timely symptom identification, accurate assessment, and appropriate management. Cardiovascular diseases account for the largest proportion (39%) of people in need of PC, and approximately one third of adults requiring PC have a cancer diagnosis [1]. Among the core competencies of PC, symptom management is particularly critical. Dyspnoea presents one of the most prevalent and distressing symptoms among patients with advanced illness, including cancer [1], with reported prevalence varying widely across studies. Earlier research, such as Solano et al. [2], estimated prevalence rates ranging from 10% to 70%, and these figures continue to be cited in more recent literature, including Hui et al. [3] and Mori et al. [4], specifically among patients with advanced cancer. Although newer literature, such as Hou et al. [5], provides updated systematic review of prevalence and factors of dyspnoea, it still relies on older data, notably from Bruera et al. [6]. Importantly, the occurrence and severity of dyspnoea among patients with advanced cancer tend to escalate during the final weeks of life [7], underscoring the need for timely recognition and disease-specific clinical decision-making from registered nurses (hereafter referred to as nurses) in PC settings.

This study forms part of a broader research project aimed at improving the recognition, assessment, and management of dyspnoea in patients with cancer receiving PC. Empirical evidence on nurses’ competence in dyspnoea care remains limited, and the available studies have primarily relied on nurses’ subjective self-evaluations [8] or instruments focusing on general PC knowledge rather than dyspnoea-specific competence [9, 10]. Based on this, it is essential that registered nurses are equipped to accurately identify dyspnoea in patients with cancer, including those whose symptoms may be less apparent or easily overlooked in everyday clinical practice. To ensure that nurses can provide high‑quality and patient‑safe care and possess the necessary knowledge to manage dyspnoea symptoms, reliable and validated instruments are needed to assess competence in specific areas of expertise. Although self‑report questionnaires offer validated tools for evaluating nurses’ self‑assessed proficiency in PC [11], considerably fewer tools have been developed to specifically assess nurses’ knowledge. Ensuring adequate competence requires systematic assessment of knowledge, and well‑constructed knowledge tests can serve as an important means of identifying knowledge gaps. Such assessment methods support the planning of targeted education and help ensure high‑quality symptom management, particularly for symptoms such as dyspnoea, which often remain under‑recognized despite their clinical significance among patients with cancer receiving PC.

Consequently, to the best of our knowledge, the assessment of nurses’ knowledge regarding dyspnoea care remains limited- not only due to the absence of a dedicated test, but also because existing tools address the topic only superficially. Although dyspnoea is included as one of the symptom domains in several assessment tools designed for nurses, no independent knowledge test specifically focused on dyspnoea care currently exists. The Palliative Care Quiz for Nursing (PCQN), a 20-item tool developed to evaluate nurses’ knowledge of PC, includes only a single pharmacological item related to dyspnoea management [12]. Similarly, in the Palliative Care Knowledge Test (PCKT), dyspnoea is included as one of five domains; however, out of the total 20 items, only four pertain to dyspnoea care, and these focus exclusively on knowledge of pharmacological interventions [13]. The most comprehensive and recent instrument is the 25-item Palliative Care Knowledge Questionnaire-Basic (PCKQ-B), a knowledge test that incorporates statements from the PCQN and PCKT tools, among others [14]. Nevertheless, it does not offer a focused or in-depth evaluation of dyspnoea care. To address this limitation, the ‘Knowledge of Dyspnoea Care of Patients in Palliative Care (DysPalKT)’ test (Copyright 2024© Kero, Koivisto, Haavisto) was developed to assess nurses’ knowledge regarding the care of dyspnoea of patients with cancer receiving PC. Advanced cancer was selected as the target diagnosis for the knowledge test because dyspnoea, alongside pain and gastrointestinal symptoms, is a common symptom among patients with cancer. However, despite its prevalence, dyspnoea has received comparatively little attention in research on symptom-management competencies among patients with cancer receiving PC. The validation of the test was conducted out as part of a digital educational intervention study targeting nurses working in non-specialist PC wards.

## Methodology

### Aim, purpose and design

The aim of this study was to develop and psychometrically test the DysPalKT. The test was specifically developed for the purpose of this study to measure nurses’ knowledge of dyspnoea care for inpatients with cancer receiving PC. The ultimate goal was to generate evidence regarding nurses’ expertise in dyspnoea care among patients with cancer receiving PC, as well as to identify nurses’ continuing education needs.

This study used a cross-sectional methodological design [15]. This study employed two phases: development and evaluation phases. During the evaluation phase, psychometric properties of the DysPalKT were assessed.

### Development phase

The development phase was conducted in three stages: the theoretical base, content validation, and pilot testing. Each stage is described in the following sections.

#### Stage one: Theoretical base

**Fig. 1:**
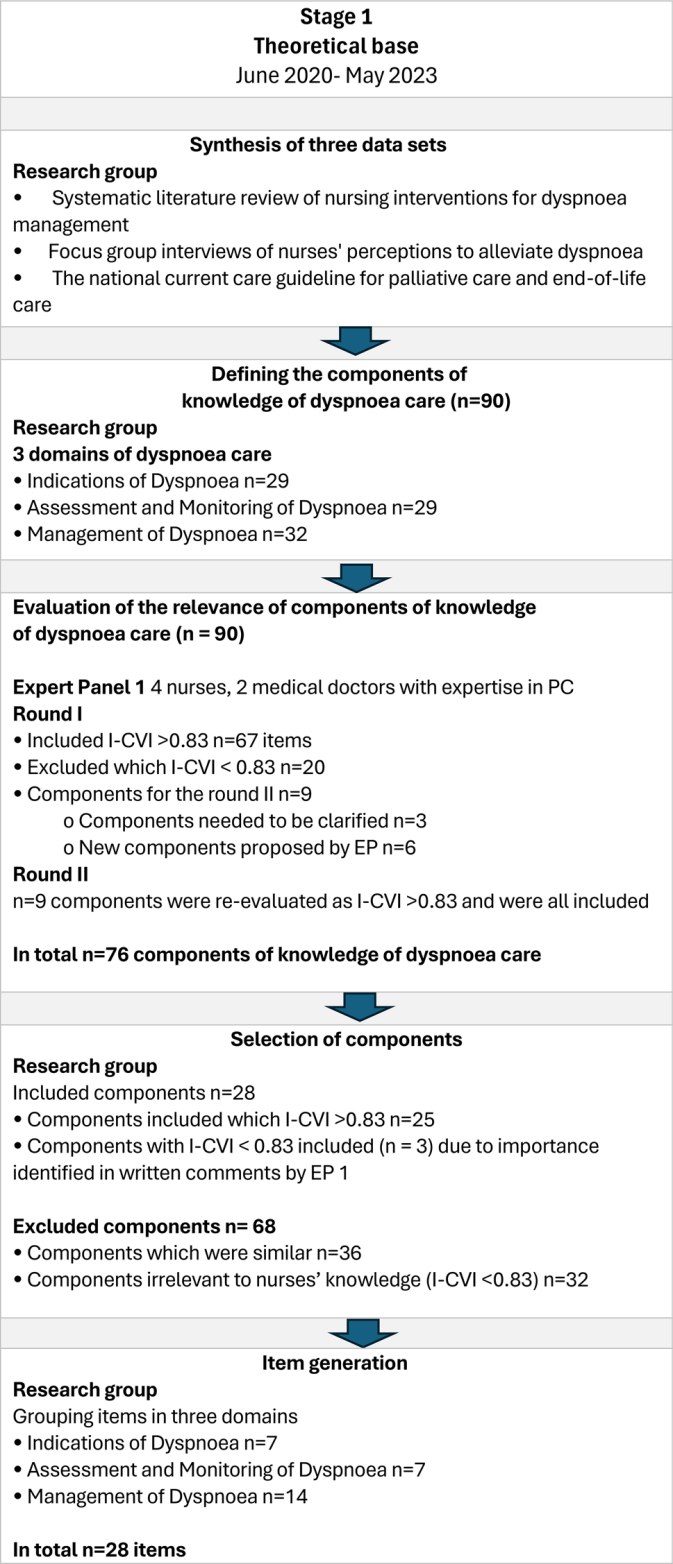
Stage 1. The theoretical base of the DysPalKT: synthesis of the data sets, defining the core components and relevance of the components

The theoretical base of the DysPalKT is based on three data sets: (1) a systematic literature review of nursing interventions for dyspnoea management [16]; (2) focus group interviews of nurses’ perceptions to alleviate dyspnoea [17]; and (3) the national current care guideline for PC [18]. The synthesis of these data sets formed the base for the content of the knowledge test for nurses. By synthesizing findings, 90 core components were identified as essential for nurses caring for patients with cancer receiving PC and experiencing dyspnoea. These core components were further categorized into three domains: 29 components addressing *Indications of Dyspnoea*, 29 focusing on *Assessment and Monitoring of Dyspnoea*, and 32 related to *Management of Dyspnoea.* (Fig. 1.)

The relevance of the components was evaluated in two rounds by Expert Panel (EP) 1, which consisted of six palliative care experts: four nurses and two medical doctors who worked on a PC ward and identified the best professional dyspnoea care competencies required of nurses when caring for a patient with cancer suffering from dyspnoea receiving PC. In this evaluation, a commonly used four-point relevance scale [originally proposed by Davis 1992], was used. The scale included the following categories: 1 = not relevant, 2 = somewhat relevant, 3 = quite relevant, and 4 = highly relevant. In the first round, components that received an item content validity index (I-CVI) score greater than 0.83 (*n* = 67) were included. Those with a score below 0.83 were excluded (*n* = 20) [19].

Additionally, three components that were identified as unclear by EP 1, as well as six new components considered important by the experts, were included in the second round of expert review by EP 1. Thus, six new and three clarified components, each representing knowledge that nurses should possess regarding the care of patients with cancer suffering from dyspnoea, were re-evaluated in this second round. After two rounds of evaluation by EP 1, the total number of components was 76.

A total of 76 components were reviewed during the component selection process. Based on the relevance evaluation conducted by Expert Panel 1 (EP1), 25 components with an item-level content validity index (I-CVI) of 0.83 or higher were selected, reflecting both their theoretical underpinnings and their importance for nurses’ knowledge in dyspnoea care. Additionally, three components with lower I-CVI values were retained, as EP1 emphasized their importance for nursing practice in written comments. In total, 68 components were excluded based on EP1’s evaluation in Stage I. Of these, 32 had an I-CVI value below 0.83 and were considered non-essential for nurses’ knowledge in the context of dyspnoea care.

In the concluding part of Stage I, the research group conducted the item generation process. A total of 28 components, each representing essential knowledge required by nurses in the care of patients with cancer suffering from dyspnoea, were transformed into test items. These items were formulated as statements with binary response options (correct/incorrect). The items were categorized into three domains: seven items addressing *Indications of Dyspnoea*, seven focusing on *Assessment and Monitoring of Dyspnoea*, and 14 related to *Management of Dyspnoea* (Fig. 1).

#### Stage two: Content validation

**Fig. 2:**
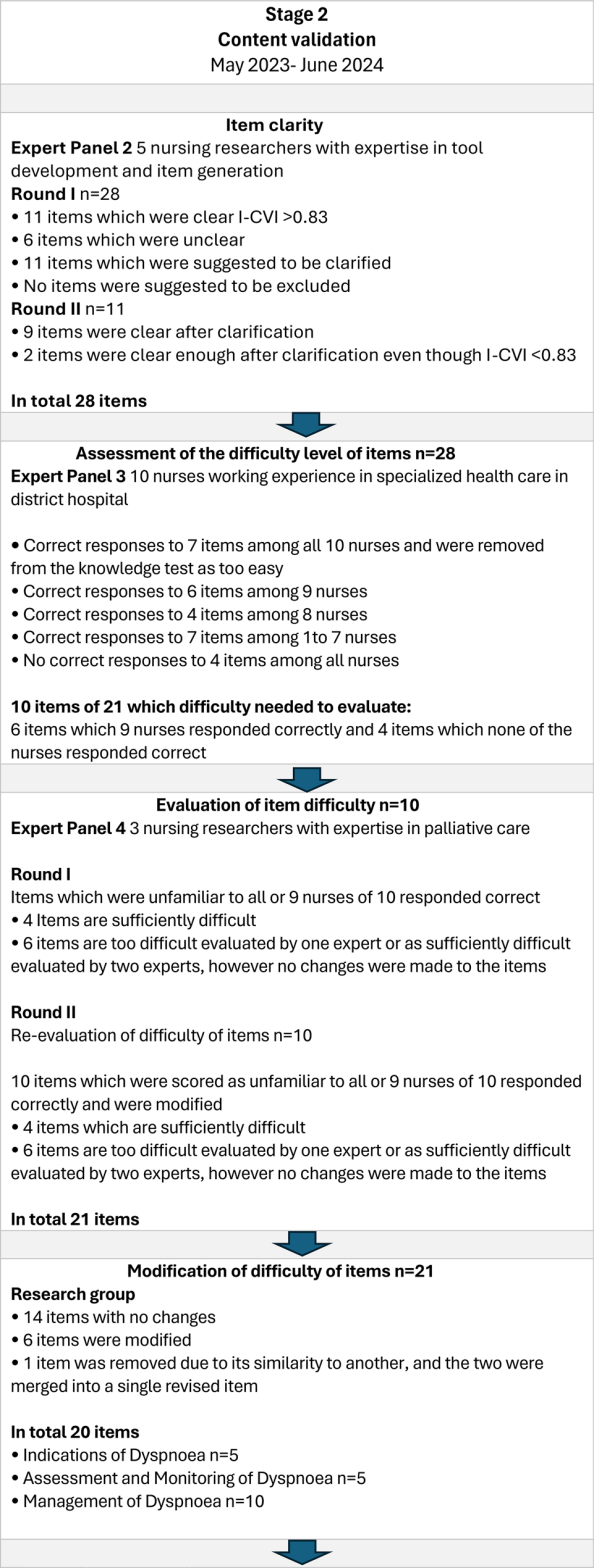
Stage 2, content validity of the instrument: modification of items, re-evaluation of difficulty of items, and second re-evaluation of items

Expert panels with distinct competencies were engaged at different stages of the test‑development process. In Stage Two, an expert panel (EP 2) comprising five nursing researchers with substantial experience in tool development and item generation evaluated the item clarity of the DysPalKT. The I-CVI was utilized to identify items with deficiencies in clarity, thereby aiming to improve the comprehensibility of the DysPalKT (see e.g. [20]. After the first round, eleven items with an I-CVI score of 0.83 or higher were approved without modifications due to their clarity. In contrast, six items were considered unclear, and eleven were suggested to be revised for improved clarity. The research group revised the items based on comments from EP 2 of Stage Two. After the second round of item revision, the clarity of all items was approved, and the number of 28 items remained unchanged (Fig. 2).

Following this, the DysPalKT underwent an assessment of difficulty level conducted by Expert Panel (EP) 3, ten nurses who had work experience in an inpatient ward not specialized in PC and cared patients with cancer receiving PC. The nurses in EP 3 were personally selected by the researcher based on direct knowledge of their clinical expertise. As they did not work in PC settings, they focused solely on evaluating the difficulty level of the items. The nurses of the EP 3 completed the electronic version of the knowledge test, which included an open-ended question at the end for written feedback. As a result, all ten nurses answered seven out of the 28 items correctly. Fourteen items were answered correctly by between one and nine nurses. None of the nurses responded correctly to the remaining four items. As written feedback, EP 3 perceived the knowledge test as easy and considered some of the items to be self-evident. Based on the assessment of item difficulty by EP 3, seven items that all nurses answered correctly were removed from the knowledge test. Of the 21 items, ten required further evaluation of difficulty: six items were answered correctly by nine nurses, indicating they may have been too easy, and four items were not answered correctly by any nurse, suggesting they may have been too difficult.

To further refine the assessment, the difficulty of ten items of 21 was evaluated based on nurses’ responses. These items, either not answered correctly by any or consistently answered correctly by nine of ten nurses, were reviewed by Expert Panel 4 (EP 4), consisting of three nursing researchers with expertise in PC. EP 4 assessed the difficulty of the items using predefined categories: “*The item is too easy*,” “*The item is sufficiently difficult*,” and “*The item is too difficult*” and provided recommendations for their revision.

Additionally, they were given the opportunity to provide suggestions for item revision. Based on EP 4’s assessment of round I, four items were considered sufficiently difficult, while the remaining six were deemed too difficult by one expert and sufficiently difficult by the other two. After the modification following the suggestion of EP 3 of round II, ten items were re-evaluated by them. As a result, altogether four items were evaluated as sufficiently difficult, and six items were evaluated by one expert as too difficult but two experts as sufficiently difficult. Consequently, these items were retained, resulting in a total of 21 items.

While EP 3 provided valuable input, it did not sufficiently support the enhancement of item difficulty at this stage of the process. The number of the items was 21 at this point. Therefore, the research group proceeded to modify the knowledge test independently. Fourteen items had no changes, six were modified and one item was removed due to its similarity to another, and the two were merged into a single revised item. At his point, the DysPalKT contained 20 items in three domains: five items addressing *Indications of Dyspnoea*, five focusing on *Assessment and Monitoring of Dyspnoea*, and ten related to *Management of Dyspnoea.*

#### Stage three: Pilot testing

**Fig. 3:**
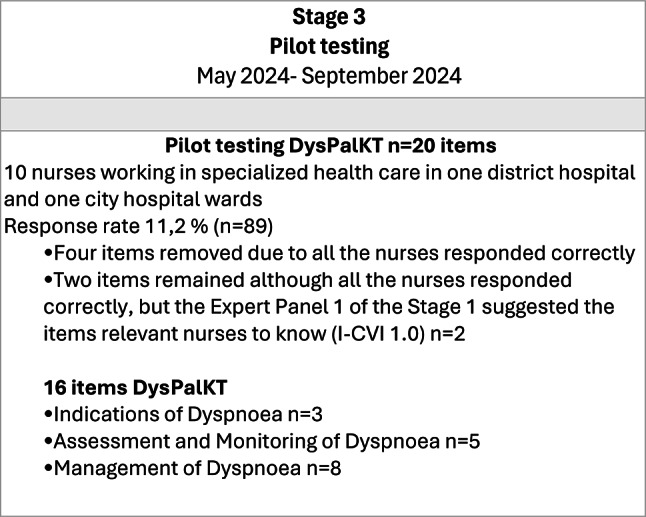
Stage 3, pilot test of the DysPalKT, and the final version with 16 items

On stage three, the DysPalKT test was piloted by nurses working in specialized health care in one district hospital and one city hospital, whose units were not specialized in PC. Of the 89 nurses working in the eligible units, ten responded to the pilot test, resulting in a response rate of 11.2%. After pilot testing, four items were removed because all ten nurses answered them correctly, indicating that the items were too easy. Two items were retained despite all nurses answering them correctly, as the Expert Panel (EP) 1 of stage one considered them essential for nurses to know (I-CVI 1.0). The final DysPalKT consist of sixteen items in three domains: three items addressing *Indications of Dyspnoea*, five focusing on *Assessment and Monitoring of Dyspnoea*, and eight related to *Management of Dyspnoea* Management. Each item includes answer options “correct”, “incorrect” or “I don’t know”. (Fig. 3).

### Evaluation phase

#### Participants and data collection

The study was conducted in general inpatient wards across seven district hospitals in Finland, where PC is provided in non-specialist settings. The inclusion criteria of nurses were current employment in eligible district hospital inpatient ward not specialized in PC, registration as a nurse, and voluntary participation. A total of 542 nurses were invited, of whom 84 participated in the study, representing approximately 15.5% of the eligible target population.

Data was collected via a cross-sectional online survey between October 2024 and March 2025. The research permits were applied from each of the seven district hospitals. Once the research permit was granted, the study protocol was presented to the chief nursing officers and head nurses of the participating wards in each district hospital prior to data collection. The head nurses of the participating wards sent invitations to participate in the study by e-mail to nurses. The invitation comprised detailed instructions regarding study participation, a statement outlining the data policy, and a digital link to access the DysPalKT. Data accumulated slowly, and the data-collection period was therefore extended to six months until March 2025. In addition, chief nursing officers and head nurses were requested to distribute the invitation several times during the data-collection period. Nevertheless, no additional nurses were recruited to complete the knowledge test.

### Statistical analysis

Two complementary approaches were used to evaluate the psychometric properties of the DysPalKT. Classical Test Theory (CTT) was applied to assess reliability, particularly suitable for the small sample size, while Item Response Theory (IRT), specifically Rasch analysis, was used to model item-level performance based on latent trait estimation [21], p. 40–41, p. 107–108).

To evaluate the psychometric properties of the DysPalKT, data were pre-processed to ensure completeness, as nurses could only proceed after answering all items. Responses were scored as + 1 (correct), 0 (uncertain), and − 1 (incorrect), with incorrect responses recoded to 0 for dichotomous analysis. Item-level correlations were analyzed using Microsoft Excel (version 2504, Build 18730.20240). Internal consistency was calculated using the KR-20 formula [22], and Rasch analysis was conducted with Winsteps v5.9.1.0 using a dichotomous model. Rating scale functioning was verified, and unidimensionality was assessed via principal component analysis of residuals, with criteria based on explained variance and eigenvalues [23]. Item fit was evaluated using MnSq values (acceptable range 0.6–1.4) and standardized z-scores [24]. Person separation and reliability were calculated to determine the test’s ability to distinguish between ability levels, with an index > 1.5 indicating sufficient differentiation [25]. Person-response validity was assessed using infit statistics, with acceptable misfit rates below 5% [26, 27]. A Wright map was constructed to visualize item difficulty and respondent ability.

### Ethics approval and consent to participate

This study was conducted in accordance with the ethical principles of responsible research conduct, including the guidelines of the National Advisory Board on Research Ethics [28] and the Declaration of Helsinki [29]. Ethical approval for conducting the study was obtained from the Ethics Committee of the Tampere Region in October 2023, approval number 148/2023. Institutional permissions were granted by the head nurses of the relevant clinical areas in all district hospitals across the wellbeing services counties. All participants were informed about the purpose of the study, its voluntary and anonymous nature, their rights as research participants, and the procedure for withdrawing from the study. Consent was given through an introductory section outlining the study’s purpose and nature. Informed consent to participate was obtained from all participants at the beginning of the online test. Only those who selected “I agree to participate in the study” were able to proceed to the test; those who selected “I do not wish to participate in the study” were not able to continue.

## Results

### Characteristics of participants

A total of 84 nurses completed DysPalKT. The demographic information for the participants is provided in Table 1. More than half of the participants (55%) were aged between 31 and 50 years, and three-quarters (75%) held a Bachelor of Health Care degree from a university of applied sciences (UAS). The most common length of professional experience as a nurse was over ten years (39%), while seven nurses (8%) had less than one year of experience. Experience specifically in PC was limited: one-third of the nurses (39%) had less than six months of experience, and only 5% had more than ten years of experience in the field. Over three-quarters (77%) of the nurses participated in the care of patients receiving PC once a month or less frequently, whereas 23% were involved in such care on a daily or weekly basis. In the nurses’ wards, the typical number of patients receiving PC was between one and five per month (68%).

**Table 1:** The demographic information of participants (*n* = 84)

Variable	*n*	%
**Age (years)**		
31–40 41–50 25–30 51–60 under 25 over 60	25	30
	21	25
	17	20
	16	19
	3	4
	2	2
**Highest nursing education**		
Bachelor of Health Care (UAS) Bachelor of Health Care Specialized Nurse Master of Health Care	63	75
	14	17
	5	6
	2	2
**Total work experience as a nurse**		
over 10 years over 5 years-10 years over 1 year-5 years 6–12 m	33	39
	27	32
	17	20
	7	8
**Work experience in palliative care**		
less than 6 m over 1 year-5 years over 5 years-10 years 6–12 m over 10 years	33	39
	29	35
	12	14
	6	7
	4	5
**How often do you care for cancer patients receiving palliative care?**		
Less than once a month Monthly Weekly Daily	33	39
	32	38
	14	17
	5	6
**How many patients receiving palliative care are cared on your ward on average per month?**		
1–2 3–5 6–10 16 or more 11–15	31	37
	26	31
	18	21
	6	7
	3	4
**Master degree in Palliative care**		
No Studying at the moment	83	99
	1	1
**Specialization studies in palliative care?**		
No Yes Studying at the moment	73	67
	9	17
	2	2
**How many continuing education courses in palliative care have you attended in the past two years?**		
0 1 3 2	56	67
	14	17
	7	8
	7	8

### Descriptive results of DysPalKT performance

The DysPalKT consists of 16 items. Only one nurse (*n* = 84) received 16 points, and one scored 0 points. More than half of the nurses (60,7%) scored between 6 and 10 points, one quarter (26,2%) achieved the highest scores (11–16 points), and slightly more than one tenth (13,1%) scored the lowest (0–5 points) (Table 1). The mean score was 8.77. (Table 2).

**Table 2:** Nurses’ knowledge of dyspnoea care in patients with cancer receiving palliative care (*n* = 84)

Score Range max. 16 points	Number of Participants (*n* = 84)	%	Mean score	SD	Median
0–5 points	11	13.1			
6–10 points	51	60.7			
11–16 points	22	26.2			
			8.77	3.64	10

The percentage of correct responses for each item (Q) is presented below in Table 3. The domain of Indications of Dyspnoea Q5 “*Constipation-induced diaphragmatic breathing difficulty can worsen dyspnoea*” had the highest proportion of correct answers (96%), while the domain Management of Dyspnoea Q6 “*Supplemental oxygen effectively relieves dyspnoea”* was the most answered incorrectly (41%).

### Psychometric properties of DysPalKT performance

#### Item-total correlation and KR-20

First, the internal consistency of the knowledge test was examined using item-total correlation. Item-total correlation is a common method for assessing instrument homogeneity, calculated between an item and the total score excluding that item. It is suggested that items with correlations below 0.20 should be removed from the instrument [30], p.196). A higher correlation (> 0.20) was observed in 13 items, indicating that it measures the same construct as the test as a whole. The domain Management of Dyspnoea Q14 “*Increasing airflow in the patient’s room is part of dyspnoea management*” demonstrated the strongest item-total correlation (0.509), which suggests that it is strongly aligned with the overall structure of the test. The three items with low correlations, such as the domain of Indications of Dyspnoea Q8 *“The patient is able to dress without difficulty despite dyspnoea”* (0.165), reflects the relative ease of the test (Table 3).

Second, also the Kuder-Richardson Formula 20 (KR-20) was used to assess internal consistency, as the knowledge test was designed to measure nurses’ knowledge using dichotomous items (i.e., responses were either correct or incorrect) [22]. The KR-20 classification indicates 0 as no reliability and 1 as best reliability, that is, the reliability of the test increases as the coefficient increases towards 1. A test is considered to be reliable if the coefficient value is more than 0.5. [31]. The KR-20 value of 0.306 suggests that all the 16 items in the knowledge test are not highly consistent: some items may not be measuring the same construct or may otherwise lack coherence. The KR-20 of the three domains did not lead to a notable improvement in the test’s reliability, suggesting that their contribution to internal consistency may be limited in this context: *Indications of Dyspnoea* and *Management of Dyspnoea* 0.277, and *Assessment and Monitoring of Dyspnoea* − 0.125 (Table 3).

**Table 3:** The results of the percentages of the right answers and the item-total correlation, the KR-20 values of all items and domains of the DysPalKT

ID	Item (*n* = 16)	% Correct	Item-Total correlation	KR-20
	**All items**			0.306
	**Indications of Dyspnoea**			0.277
Q5	Constipation-induced diaphragmatic breathing difficulty can worsen dyspnoea.	96	0.185	
Q8	The patient is able to dress without difficulty despite dyspnoea.	86	0.165	
Q9	Sinus tachycardia may be caused by dyspnoea.	82	0.240	
	**Assessment and Monitoring of Dyspnoea**			**-0.125**
Q1	The Edmonton Symptom Assessment System (ESAS/ESASr) is used in nursing to assess dyspnoea by both the patient and family members.	45	0.400	
Q2	When assessing the severity of dyspnoea, the patient’s own experience is primary.	94	0.307	
Q10	When a patient experiences dyspnoea, it is sufficient to monitor oxygen saturation, heart rate, and respiratory rate.	89	0.290	
Q11	The patient’s own assessment of dyspnoea is more important than the nurse’s objective evaluation.	77	0.181	
Q12	The Numerical Rating Scale (NRS) is based on an objective assessment of dyspnoea severity.	48	0.322	
	**Management of Dyspnoea**			**0.277**
Q3	Dyspnoea can be treated with opioids.	89	0.354	
Q4	Opioids reduce the need for psychosocial support.	89	0.225	
Q6	Supplemental oxygen effectively relieves dyspnoea.	41	0.359	
Q7	Pharmacological treatment is always the primary method for managing dyspnoea.	86	0.297	
Q13	Pleural puncture relieves dyspnoea by increasing lung ventilation capacity.	77	0.324	
Q14	Increasing airflow in the patient’s room is part of dyspnoea management.	80	0.501	
Q15	Palliative sedation is used in the treatment of refractory dyspnoea.	84	0.368	
Q16	The nurse plans and makes decisions regarding palliative sedation together with the patient and their family.	76	0.200	

#### Rasch analysis

Majority of responses (77%) were in category “1”. Unidimensionality was 24.8%, indicating that the test does not entirely measure single latent construct. Similarly, the explanation level of first, second, third, fourth and fifth contrasting construct indicate (8.5%, 7.7%, 7.4%, 6.8%, and 5.9%, respectively) that the scale might have multidimensional features. The eigenvalues of the contrasting constructs were 1.8, 1.6, 1.6, 1.5, and 1.3, respectively. This indicates that approximately two items per construct are measuring alternative aspects. However, the item infit was acceptable in each item (Table 4). MnSq values in each item ranged from 0.86 to 1.09.

Regarding person-response validity, person separation was low (0.58) demonstrating test’s limited ability to distinguish to at least two groups. However, person misfit was rather acceptable. There were only six nurses (7%) who had misfit values higher than 1.4.

**Table 4:** Item difficulty, standard error and infit statistics of the DysPalKT

Item			*Infit*		*Outfit*	
	Measure logits	Standard error (SE)	MnSq	Zstd	MnSq	Zsdt
11	0.24	0.28	1.09	0.69	1.49	1.97
16	0.31	0.27	1.09	0.71	1.19	0.92
8	-0.38	0.32	1.07	0.39	1.11	0.42
12	1.76	0.24	1.07	0.85	1.07	0.70
6	2.10	0.24	0.99	-0.11	1.06	0.55
9	-0.09	0.30	1.03	0.24	1.04	0.23
4	-0.73	0.36	1.01	0.13	0.89	-0.16
13	0.24	0.28	1.00	0.02	0.97	-0.04
7	-0.38	0.32	0.99	0.01	0.87	-0.30
10	-0.73	0.36	0.99	0.03	0.71	-0.65
5	-1.95	0.59	0.98	0.13	0.85	0.06
1	1.87	0.24	0.95	-0.59	0.96	-0.38
2	-1.40	0.47	0.94	-0.02	0.60	-0.61
15	-0.28	0.31	0.94	-0.23	0.77	-0.70
3	-0.73	0.36	0.93	-0.20	0.76	-0.52
14	0.16	0.28	0.86	-0.97	0.90	-0.37

**Fig. 4:**
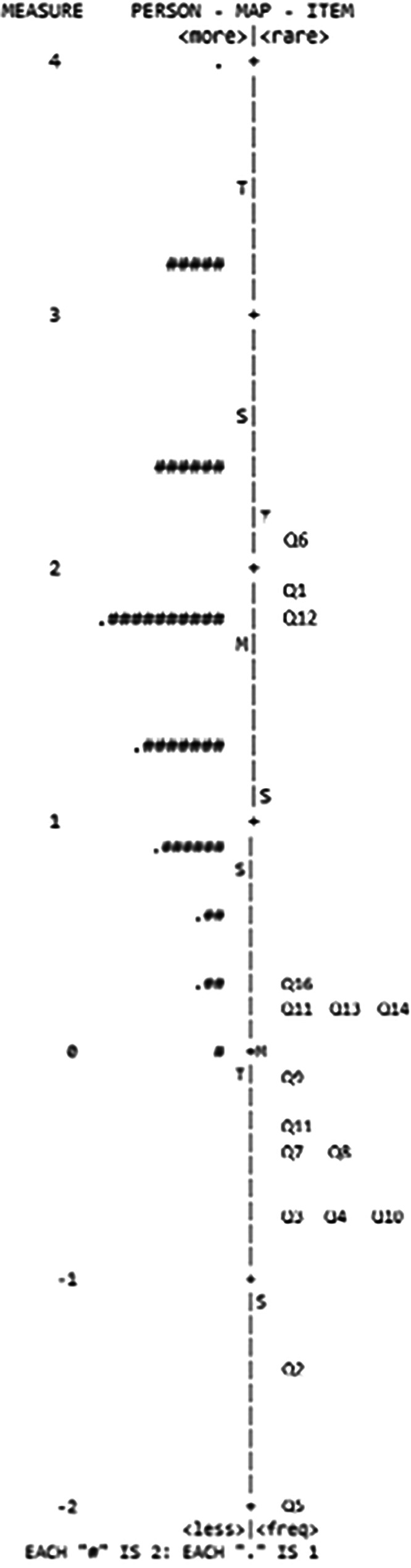
The Wright map of the DysPalKT

The Wright map demonstrated the majority of the items were too easy for the nurses (Fig. 4). There were nine items that were passed by all the nurses. Item 6 seemed to be the most difficult item but it was the majority of the nurses were able to pass that also.

## Discussion

This study presents the development and evaluation of the DysPalKT, a test designed to assess nurses’ knowledge of dyspnoea care for patients with cancer receiving PC. The knowledge test was developed in two phases: a development phase comprising theoretical synthesis, expert panel reviews, and pilot testing, and an evaluation phase.

Nurses often develop competence in PC through hands-on experience and by learning from more experienced colleagues [32]. Continuous professional development is particularly important in environments where PC is not routinely practiced, as previously acquired skills may deteriorate over time. However, the effectiveness of PC education may be questioned if nurses’ knowledge is not systematically assessed.

The DysPalKT was tested with 84 nurses with a relatively high level of clinical experience and theoretical knowledge. Despite this, knowledge gaps were evident. For example, only 41% correctly answered the item on supplemental oxygen use. Similarly, fewer than half answered items related to dyspnoea assessment tools (ESAS/ESASr and NRS) correctly. However, although the ESAS tool is globally implemented and widely used among patients in PC settings such as wards specialized in PC [33], this study demonstrates that nurses working in non-specialist wards do not routinely use symptom assessment tools such as ESAS or the NRS when evaluating the symptoms of patients receiving PC. Interestingly, while 94% of nurses correctly answered to the domain of Assessment and Monitoring of Dyspnoea Q2 (*‘When assessing the severity of dyspnoea*,* the patient’s own experience is primary*’), 77% responded correctly to the closely related to the same domain Q11 (*‘The patient’s own assessment of dyspnoea is more important than the nurse’s objective evaluation’*) revealing a 17% discrepancy in responses within the same domain.

To ensure comprehensive psychometric evaluation, both Classical Test Theory (KR-20) and Item Response Theory (Rasch analysis) were applied to assess internal consistency and item-level performance. Classical Test Theory (CTT) and Item Response Theory (IRT) offer different yet complementary perspectives on test performance. In this evaluation, CTT assessed the DysPalKT at the total-score level, producing sample-dependent results, while IRT modeled how respondents interacted with individual items in relation to their ability level and item characteristics. CTT assumes that measurement error is independent of the true score, meaning that the error variance should remain constant across respondents. However, the first version of the DysPalKT demonstrated weaker reliability among both low- and high-performing respondents, suggesting that this assumption was not fully met [34]. Additionally, the KR-20 coefficient (0.306) indicated weak internal consistency [28], likely due to the limited variability in responses and the large number of easy items, both common features of knowledge tests that can reduce internal consistency [35].

Because the test was also examined using IRT, which enables forms of reliability assessment that cannot be performed within the framework of CTT, the logistic Rasch model applied to the DysPalKT evaluated model fit by estimating the relationship between respondent ability and item difficulty, and by using infit and outfit statistics to assess the adequacy of fit to the data [34]. Overall, nurses demonstrated a good level of knowledge, which was reflected in the Rasch analysis showing that the items were predominantly easy and therefore had limited discriminatory power. Evidence of multidimensionality was also identified, and person separation remained low. In addition, nine of the 16 item thresholds clustered below zero on Wright’s map, making it difficult to distinguish between low and high performers [25].

The DyspalKT knowledge test was based on dichotomous response options. Therefore, although construct validity assessment is important, traditional exploratory factor analysis (EFA) is not optimal for dichotomous knowledge items due to violations of normality and linearity assumptions [36];[37]. The limited variability inherent in yes-no response options affect correlations and may introduce a substantial amount of non-random measurement error [36]. Therefore, two complementary approaches were applied to evaluate the reliability and structure of the first test version.

Taking together, as no comparable knowledge test currently exists, direct comparisons with previous studies are not feasible. Many nurses demonstrated ability levels exceeding the difficulty of the available items, indicating a lack of sufficiently challenging content. It should be noted that the DysPalKT was primarily completed by nurses with experience in PC, which may have limited the statistical differentiation between nurses. Nevertheless, despite their experience in PC, gaps in knowledge were identified, indicating a need for updating education in the future. Patients in PC are cared for across different levels of the healthcare system, and all healthcare professionals should possess adequate basic competence in the care of patients receiving PC. Since the development process involved multidisciplinary experts in PC who were likewise unable to increase the difficulty level of the test, these findings highlight the need to test the DysPalKT among a larger and more diverse sample, such as nursing students and primary health care settings, to support further development. For example, mastering the use of the ESAS tool is crucial, as the use of ESAS has the potential to improve patients’ quality of life and to strengthen the patient‑centeredness of PC delivery [33]. This includes refining item formulations and removing poorly correlating items to improve the test’s validity and its ability to distinguish between low- and high-performing nurses, thereby enabling a more accurate assessment of nurses’ knowledge and competence in dyspnoea care [35]. Moreover, the test has identified the basic knowledge required of nurses regarding the dyspnoea care. In the further development of the test, it will be necessary to add more advanced items in order to enable a comprehensive assessment of nurses’ level of knowledge of dyspnoea care. Future research with larger samples should employ Item Factor Analysis or IRT approaches (e.g., 2- parameter logistic models) to more rigorously examine the latent structure of the instrument.

### Limitations

Despite the study’s contributions, limitations must be acknowledged. The sample size was relatively small (*n* = 84), and the DysPalKT was tested for the first time. The low participation rate may have introduced selection bias, limiting generalizability. The number of participating nurses did not increase, even though the study’s participation period within the units was extended to six months. External factors such as staff shortages and organizational restructuring during data collection may have further impacted participation. Concurrently, mandatory online training for staff, and other ongoing studies in the wards further constrained staff availability and engagement.

## Conclusions

The DysPalKT demonstrated promising features, including acceptable item infit values and strong theoretical grounding supported by expert validation (I-CVI ≥ 0.83). However, its overall reliability was below the recommended threshold, and Rasch analysis revealed multidimensionality and limited discriminatory capacity. Although the items of the first version of the DysPalKT knowledge test did not yet form a clinical and statistical construct and the validity of the test proved inadequate [38], the test nevertheless contains several promising features that remain potentially useful. Enhancing the knowledge test could improve its capacity to identify learning needs and guide future educational interventions in non-specialist PC. Further research is warranted to evaluate its applicability across varied clinical settings and larger nurse populations, thereby strengthening its role in assessing dyspnoea care knowledge of nurses.

## Supplementary Information

Below is the link to the electronic supplementary material.

Supplementary Material 1

## Data Availability

The datasets used and/or analysed during the current study are available from the corresponding author on reasonable request.

## Abbreviations

PC: Palliative Care
CTT: Classical Test Theory
IRT: Item Response Theory
KR-20: Kuder-Richardson Formula 20
I-CVI: Item-level Content Validity Index
MnSq: Mean Square
SD: Standard Deviation
NRS: Numerical Rating Scale
ESAS: Edmonton Symptom Assessment System
EFA: Exploratory Factor Analysis
