# Supplementary material for: Development and psychometric evaluation of the DysPalKT for assessing nurses’ knowledge of dyspnoea care in patients with cancer receiving palliative care
Source: BMC Nurs. 2026 Mar 14;25:386. doi: 10.1186/s12912-026-04540-z (PMC13104488; doi:10.1186/s12912-026-04540-z)
Supplement: Supplementary file 1 — Supplementary Material 1 [file 12912_2026_4540_MOESM1_ESM.pdf]

Knowledge of Dyspnoea Care of Patients in Palliative Care (DysPalKT)

Copyright 2024© Kero, Koivisto, Haavisto

1. The Edmonton Symptom Assessment System (ESAS/ESASr) is used in nursing to assess dyspnoea by both the patient and family members.	Yes<br>No<br>I don't know
2. When assessing the severity of dyspnoea, the patient's own experience is primary.	Yes<br>No<br>I don't know
3. Dyspnoea can be treated with opioids.	Yes<br>No<br>I don't know
4. Opioids reduce the need for psychosocial support.	Yes<br>No<br>I don't know
5. Constipation-induced diaphragmatic breathing difficulty can worsen dyspnoea.	Yes<br>No<br>I don't know
6. Supplemental oxygen effectively relieves dyspnoea.	Yes<br>No<br>I don't know
7. Pharmacological treatment is always the primary method for managing dyspnoea.	Yes<br>No<br>I don't know
8. The patient is able to dress without difficulty despite dyspnoea.	Yes<br>No<br>I don't know
9. Sinus tachycardia may be caused by dyspnoea.	Yes<br>No<br>I don't know
10. When a patient experiences dyspnoea, it is sufficient to monitor oxygen saturation, heart rate, and respiratory rate.	Yes<br>No<br>I don't know
11. The patient's own assessment of dyspnoea is more important than the nurse's objective evaluation.	Yes<br>No<br>I don't know
12. The Numerical Rating Scale (NRS) is based on an objective assessment of dyspnoea severity.	Yes<br>No<br>I don't know
13. Pleural puncture relieves dyspnoea by increasing lung ventilation capacity.	Yes<br>No<br>I don't know
14. Increasing airflow in the patient's room is part of dyspnoea management.	Yes<br>No<br>I don't know
15. Palliative sedation is used in the treatment of refractory dyspnoea.	Yes<br>No<br>I don't know
16. The nurse plans and makes decisions regarding palliative sedation together with the patient and their family.	Yes<br>No<br>I don't know
